# 2-Amino-6-methyl­pyridinium 3-chloro­benzoate

**DOI:** 10.1107/S1600536813002559

**Published:** 2013-02-02

**Authors:** Kaliyaperumal Thanigaimani, Nuridayanti Che Khalib, Suhana Arshad, Ibrahim Abdul Razak

**Affiliations:** a School of Physics, Universiti Sains Malaysia, 11800 USM, Penang, Malaysia

## Abstract

In the title salt, C_6_H_9_N_2_^+^·C_7_H_4_ClO_2_^−^, the 3-chloro­benzoate anion shows a whole-mol­ecule disorder over two positions with a refined occupancy ratio of 0.505 (4):0.495 (4). In the crystal, the cations and anions are linked *via* N—H⋯O hydrogen bonds, forming a centrosymmetric 2 + 2 aggregate with *R*_2_^2^(8) and *R*_4_^2^(8) ring motifs. The crystal structure also features a π–π stacking inter­action between the pyridinium rings with a centroid–centroid distance of 3.8339 (9) Å.

## Related literature

For background to the chemistry of substituted pyridines, see: Pozharski *et al.* (1997 ▶); Katritzky *et al.* (1996 ▶). For related structures, see: Hemamalini & Fun (2010 ▶); Thanigaimani *et al.* (2012 ▶); Draguta *et al.* (2012 ▶). For hydrogen-bond motifs, see: Bernstein *et al.* (1995 ▶). For bond-length data, see: Allen *et al.* (1987 ▶). For stability of the temperature controller used for the data collection, see: Cosier & Glazer (1986 ▶).
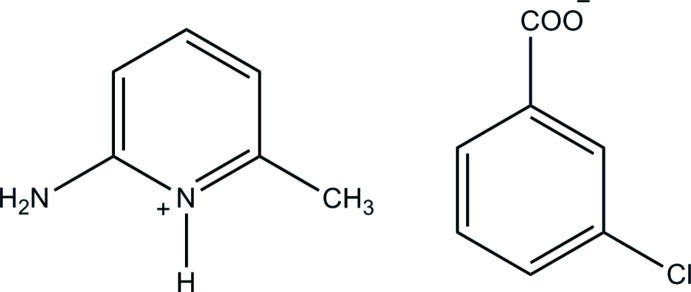


## Experimental

### 

#### Crystal data

C_6_H_9_N_2_^+^·C_7_H_4_ClO_2_^−^
*M* *_r_* = 264.70
Monoclinic, 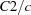

*a* = 22.3118 (15) Å
*b* = 15.2053 (10) Å
*c* = 7.4166 (5) Å
β = 100.924 (1)°
*V* = 2470.5 (3) Å^3^
*Z* = 8
Mo *K*α radiation
μ = 0.30 mm^−1^
*T* = 100 K
0.36 × 0.06 × 0.05 mm

#### Data collection

Bruker SMART APEXII DUO CCD area-detector diffractometer
Absorption correction: multi-scan (*SADABS*; Bruker, 2009 ▶) *T*_min_ = 0.898, *T*_max_ = 0.985
24731 measured reflections
3575 independent reflections
2446 reflections with *I* > 2σ(*I*)
*R*_int_ = 0.053

#### Refinement

*R*[*F* ^2^ > 2σ(*F* ^2^)] = 0.045
*wR*(*F* ^2^) = 0.128
*S* = 1.06
3575 reflections
267 parameters
5 restraints
H atoms treated by a mixture of independent and constrained refinement
Δρ_max_ = 0.29 e Å^−3^
Δρ_min_ = −0.28 e Å^−3^

### 

Data collection: *APEX2* (Bruker, 2009 ▶); cell refinement: *SAINT* (Bruker, 2009 ▶); data reduction: *SAINT*; program(s) used to solve structure: *SHELXTL* (Sheldrick, 2008 ▶); program(s) used to refine structure: *SHELXTL*; molecular graphics: *SHELXTL*; software used to prepare material for publication: *SHELXTL* and *PLATON* (Spek, 2009 ▶).

## Supplementary Material

Crystal structure: contains datablock(s) global, I. DOI: 10.1107/S1600536813002559/is5234sup1.cif

Structure factors: contains datablock(s) I. DOI: 10.1107/S1600536813002559/is5234Isup2.hkl

Supplementary material file. DOI: 10.1107/S1600536813002559/is5234Isup3.cml

Additional supplementary materials:  crystallographic information; 3D view; checkCIF report

## Figures and Tables

**Table 1:** Hydrogen-bond geometry (Å, °)

*D*—H⋯*A*	*D*—H	H⋯*A*	*D*⋯*A*	*D*—H⋯*A*
N1—H2*N*2⋯O1	0.94 (2)	1.69 (2)	2.614 (7)	168 (2)
N2—H1*N*2⋯O2	0.86 (2)	1.98 (3)	2.832 (14)	170 (2)
N2—H1*N*1⋯O2^i^	0.88 (2)	2.06 (2)	2.853 (11)	150 (2)

Symmetry code: (i) 
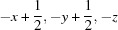
.

**Fig. 1.**
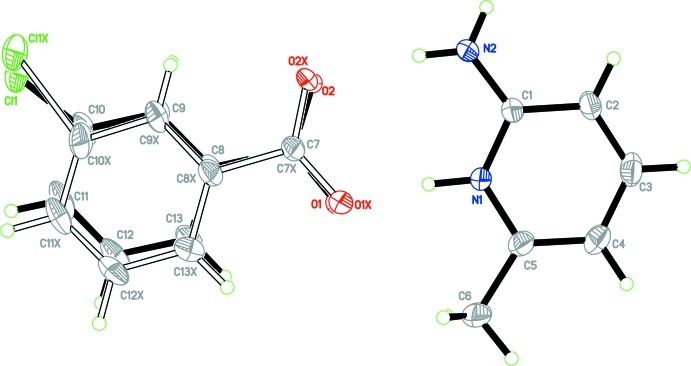
The molecular structure of the title compound with atom labels with 50% probability displacement ellipsoids. All disordered components are shown.

**Fig. 2.**
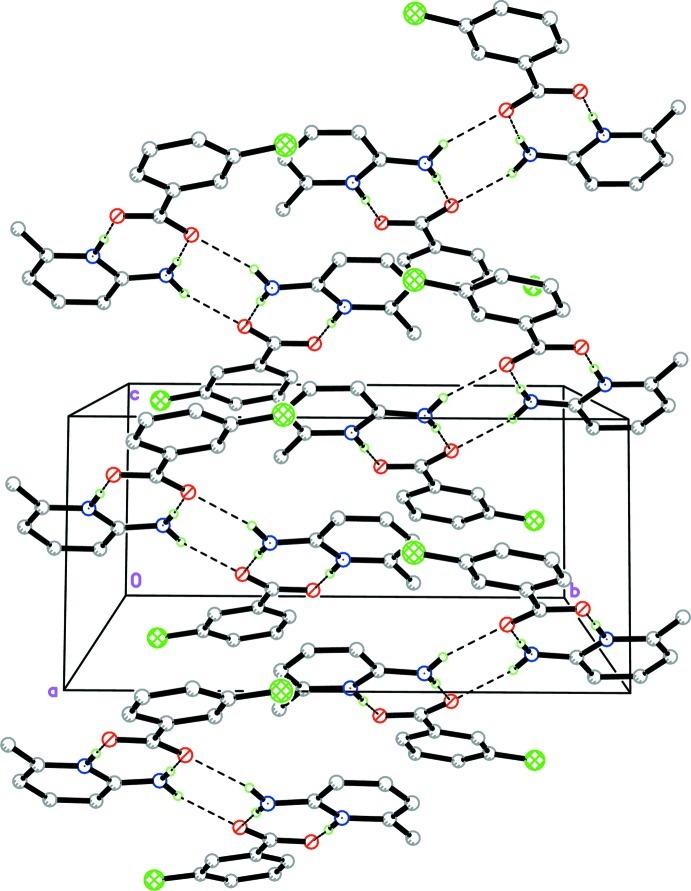
The crystal packing of the title compound. Only major disordered component is shown. H atoms not involved in the hydrogen bonds (dashed lines) have been omitted for clarity.

## supplementary crystallographic information

### Comment

Pyridine and its derivatives play an important role in heterocyclic chemistry (Pozharski *et al.*, 1997; Katritzky *et al.*, 1996). Related crystal structures of 2-amino-4-methylpyridinium 3-chlorobenzoate (Hemamalini & Fun, 2010) and 2-amino-5-methylpyridinium 3-chlorobenzoate (Thanigaimani *et al.*, 2012) have been reported. In order to study potential hydrogen bonding interactions the crystal structure determination of the title compound (I) was carried out.

The asymmetric unit (Fig. 1) contains one 2-amino-6-methylpyridinium cation and one 3-chlorobenzoate anion. The proton transfers from the one of the carboxyl group oxygen atom (O1) to atom N1 of 2-amino-5-methylpyrimidine resulted in the widening of C1—N1—C5 angle of the pyridinium ring to 122.93 (13)°, compared to the corresponding angle of 118.43 (9)° in neutral 6-methylpyridin-2-amine (Draguta *et al.*, 2012). The whole 3-chlorobenzoate anion is disordered over two positions with a refined occupancy ratio of 0.505 (4):0.495 (4). The 2-amino-5-methylpyridinium cation is essentially planar, with a maximum deviation of 0.030 (2) Å for atom C4. The bond lengths (Allen *et al.*, 1987) and angles are normal.

In the crystal packing (Fig. 2), the protonated N1 atom and the 2-amino group (N2) are hydrogen-bonded to the carboxylate oxygen atoms (O1 and O2) *via* a pair of intermolecular N1—H2N2···O1 and N2—H1N2···O2 hydrogen bonds, forming an *R*_2_^2^(8) (Bernstein *et al.*, 1995) ring motif. The motifs are centrosymmetrically paired *via* intermolecular N2—H1N1···O2^i^ hydrogen bonds between the ions which form *R*_4_^2^(8) ring motif, resulting in a DDAA array (where D is a hydrogen-bond donor and A is a hydrogen-bond acceptor) of quadruple hydrogen bonds (symmetry code in Table 1). The crystal structure is further stabilized by a π–π interaction between the pyridinium ring (*Cg*1 = N1/C1–C5) cations [*Cg*1···*Cg*1 = 3.8339 (9) Å; *x*, 1 - *y*, -1/2 - *z*].

### Experimental

Hot methanol solutions (20 ml) of 2-amino-6-methylpyridine (54 mg, Aldrich) and 3-chlorobenzoic acid (39 mg, Merck) were mixed and warmed over a heating magnetic stirrer hotplate for a few minutes. The resulting solution was allowed to cool slowly at room temperature and crystals of the title compound (I) appeared after a few days.

### Refinement

N-bound H atoms were located in a difference Fourier map and refined freely [refined N—H distances: 0.94 (2), 0.88 (2) and 0.86 (2) Å]. The remaining H atoms were positioned geometrically (C—H = 0.95 and 0.98 Å) and were refined using a riding model, with *U*_iso_(H) = 1.2*U*_eq_(C) or 1.5*U*_eq_(methyl C). A rotating-group model was used for the methyl group. The whole 3-chlorobenzoate anion was disordered over two positions with a refined ratio of 0.505 (4):0.495 (4). For the disordred anion, bond-length restraints [Cl—C = 1.73 (1) Å and O—C = 1.23 (1) Å] and a *SAME* instruction were applied.

### Crystal data

**Table:**

C_6_H_9_N_2_^+^·C_7_H_4_ClO_2_^−^	*F*(000) = 1104
*M**_r_* = 264.70	*D*_x_ = 1.423 Mg m^−^^3^
Monoclinic, *C*2/*c*	Mo *K*α radiation, λ = 0.71073 Å
Hall symbol: -C 2yc	Cell parameters from 4651 reflections
*a* = 22.3118 (15) Å	θ = 2.7–29.3°
*b* = 15.2053 (10) Å	µ = 0.30 mm^−^^1^
*c* = 7.4166 (5) Å	*T* = 100 K
β = 100.924 (1)°	Needle, colourless
*V* = 2470.5 (3) Å^3^	0.36 × 0.06 × 0.05 mm
*Z* = 8	

### Data collection

**Table:**

Bruker SMART APEXII DUO CCD area-detector diffractometer	3575 independent reflections
Radiation source: fine-focus sealed tube	2446 reflections with *I* > 2σ(*I*)
Graphite monochromator	*R*_int_ = 0.053
φ and ω scans	θ_max_ = 30.0°, θ_min_ = 1.6°
Absorption correction: multi-scan (*SADABS*; Bruker, 2009)	*h* = −30→31
*T*_min_ = 0.898, *T*_max_ = 0.985	*k* = −21→21
24731 measured reflections	*l* = −10→10

### Refinement

**Table:**

Refinement on *F*^2^	Primary atom site location: structure-invariant direct methods
Least-squares matrix: full	Secondary atom site location: difference Fourier map
*R*[*F*^2^ > 2σ(*F*^2^)] = 0.045	Hydrogen site location: inferred from neighbouring sites
*wR*(*F*^2^) = 0.128	H atoms treated by a mixture of independent and constrained refinement
*S* = 1.06	*w* = 1/[σ^2^(*F*_o_^2^) + (0.0584*P*)^2^ + 1.2878*P*] where *P* = (*F*_o_^2^ + 2*F*_c_^2^)/3
3575 reflections	(Δ/σ)_max_ < 0.001
267 parameters	Δρ_max_ = 0.29 e Å^−^^3^
5 restraints	Δρ_min_ = −0.28 e Å^−^^3^

### Special details

**Table:**

Experimental. The crystal was placed in the cold stream of an Oxford Cryosystems Cobra open-flow nitrogen cryostat (Cosier & Glazer, 1986) operating at 100.0 (1) K.
Geometry. All e.s.d.'s (except the e.s.d. in the dihedral angle between two l.s. planes) are estimated using the full covariance matrix. The cell e.s.d.'s are taken into account individually in the estimation of e.s.d.'s in distances, angles and torsion angles; correlations between e.s.d.'s in cell parameters are only used when they are defined by crystal symmetry. An approximate (isotropic) treatment of cell e.s.d.'s is used for estimating e.s.d.'s involving l.s. planes.
Refinement. Refinement of *F*^2^ against ALL reflections. The weighted *R*-factor *wR* and goodness of fit *S* are based on *F*^2^, conventional *R*-factors *R* are based on *F*, with *F* set to zero for negative *F*^2^. The threshold expression of *F*^2^ > σ(*F*^2^) is used only for calculating *R*-factors(gt) *etc*. and is not relevant to the choice of reflections for refinement. *R*-factors based on *F*^2^ are statistically about twice as large as those based on *F*, and *R*- factors based on ALL data will be even larger.

### Fractional atomic coordinates and isotropic or equivalent isotropic displacement parameters (Å^2^)

**Table:**

	*x*	*y*	*z*	*U*_iso_*/*U*_eq_	Occ. (<1)
Cl1	0.51442 (12)	0.09433 (16)	0.5251 (4)	0.0377 (5)	0.505 (4)
O1	0.3604 (3)	0.4344 (4)	0.2546 (10)	0.0298 (12)	0.505 (4)
O2	0.3335 (7)	0.2966 (7)	0.1833 (12)	0.0279 (16)	0.505 (4)
C7	0.3716 (3)	0.3536 (4)	0.2547 (8)	0.0223 (11)	0.505 (4)
C8	0.43409 (15)	0.3242 (2)	0.3507 (6)	0.0224 (7)	0.505 (4)
C9	0.4472 (5)	0.2368 (8)	0.3839 (11)	0.0217 (13)	0.505 (4)
H9A	0.4166	0.1945	0.3394	0.026*	0.505 (4)
C10	0.5042 (5)	0.2072 (5)	0.4815 (14)	0.0368 (18)	0.505 (4)
C11	0.54930 (16)	0.2695 (3)	0.5297 (6)	0.0348 (9)	0.505 (4)
H11A	0.5890	0.2515	0.5885	0.042*	0.505 (4)
C12	0.53779 (14)	0.3580 (3)	0.4939 (5)	0.0345 (9)	0.505 (4)
H12A	0.5693	0.4000	0.5301	0.041*	0.505 (4)
C13	0.48029 (14)	0.3853 (2)	0.4052 (5)	0.0286 (8)	0.505 (4)
H13A	0.4725	0.4460	0.3816	0.034*	0.505 (4)
Cl1X	0.52750 (12)	0.11634 (16)	0.5138 (4)	0.0336 (4)	0.495 (4)
O1X	0.3491 (3)	0.4332 (4)	0.3044 (10)	0.0294 (11)	0.495 (4)
O2X	0.3302 (7)	0.2910 (8)	0.2304 (12)	0.0273 (15)	0.495 (4)
C7X	0.3620 (3)	0.3525 (4)	0.3103 (9)	0.0225 (11)	0.495 (4)
C8X	0.42352 (17)	0.3289 (2)	0.4266 (6)	0.0222 (8)	0.495 (4)
C9X	0.4418 (5)	0.2415 (8)	0.4305 (11)	0.0223 (14)	0.495 (4)
H9XA	0.4152	0.1963	0.3748	0.027*	0.495 (4)
C10X	0.5005 (5)	0.2239 (4)	0.5195 (13)	0.0314 (16)	0.495 (4)
C11X	0.53950 (16)	0.2854 (3)	0.6185 (6)	0.0319 (9)	0.495 (4)
H11B	0.5787	0.2691	0.6844	0.038*	0.495 (4)
C12X	0.51893 (15)	0.3713 (2)	0.6175 (5)	0.0303 (8)	0.495 (4)
H12B	0.5445	0.4155	0.6820	0.036*	0.495 (4)
C13X	0.46134 (15)	0.3931 (2)	0.5228 (5)	0.0272 (8)	0.495 (4)
H13B	0.4474	0.4522	0.5233	0.033*	0.495 (4)
N1	0.25283 (5)	0.49267 (8)	0.09853 (17)	0.0197 (3)	
N2	0.21460 (7)	0.35423 (9)	0.0235 (2)	0.0337 (4)	
C1	0.20717 (6)	0.44124 (10)	0.0094 (2)	0.0211 (3)	
C2	0.15454 (7)	0.48210 (10)	−0.0898 (2)	0.0238 (3)	
H2A	0.1223	0.4479	−0.1572	0.029*	
C3	0.15048 (7)	0.57153 (11)	−0.0877 (2)	0.0307 (4)	
H3A	0.1149	0.5996	−0.1533	0.037*	
C4	0.19795 (8)	0.62255 (11)	0.0095 (3)	0.0363 (4)	
H4A	0.1944	0.6848	0.0121	0.044*	
C5	0.24940 (7)	0.58195 (10)	0.1006 (2)	0.0257 (3)	
C6	0.30466 (8)	0.62859 (11)	0.2048 (3)	0.0372 (4)	
H6A	0.2955	0.6913	0.2136	0.056*	
H6B	0.3388	0.6213	0.1402	0.056*	
H6C	0.3157	0.6036	0.3284	0.056*	
H1N1	0.1883 (11)	0.3210 (14)	−0.050 (3)	0.046 (6)*	
H2N2	0.2887 (10)	0.4672 (15)	0.165 (3)	0.050 (6)*	
H1N2	0.2487 (11)	0.3311 (14)	0.077 (3)	0.044 (6)*	

### Atomic displacement parameters (Å^2^)

**Table:**

	*U*^11^	*U*^22^	*U*^33^	*U*^12^	*U*^13^	*U*^23^
Cl1	0.0260 (10)	0.0318 (11)	0.0523 (8)	0.0162 (6)	0.0001 (7)	0.0009 (8)
O1	0.016 (2)	0.0172 (13)	0.051 (4)	−0.0007 (13)	−0.0063 (17)	−0.0018 (18)
O2	0.020 (2)	0.0134 (18)	0.043 (4)	0.0000 (14)	−0.014 (3)	0.000 (3)
C7	0.012 (2)	0.0175 (16)	0.035 (3)	−0.0021 (13)	−0.0015 (19)	0.0004 (19)
C8	0.0103 (15)	0.0277 (16)	0.027 (2)	0.0008 (11)	−0.0011 (13)	−0.0050 (15)
C9	0.014 (2)	0.0240 (19)	0.028 (4)	0.0072 (15)	0.006 (3)	−0.005 (3)
C10	0.013 (2)	0.037 (3)	0.055 (4)	0.016 (2)	−0.006 (2)	−0.016 (2)
C11	0.0138 (16)	0.059 (3)	0.029 (2)	0.0094 (15)	−0.0032 (14)	−0.0084 (17)
C12	0.0134 (15)	0.054 (2)	0.0331 (19)	−0.0063 (14)	−0.0032 (12)	−0.0057 (15)
C13	0.0182 (15)	0.0351 (17)	0.0299 (18)	−0.0033 (12)	−0.0019 (12)	−0.0044 (13)
Cl1X	0.0247 (9)	0.0289 (10)	0.0442 (7)	0.0119 (6)	−0.0011 (6)	0.0025 (7)
O1X	0.021 (3)	0.0190 (14)	0.043 (3)	−0.0005 (14)	−0.0066 (16)	−0.0035 (17)
O2X	0.0172 (18)	0.020 (2)	0.038 (4)	−0.0025 (14)	−0.011 (3)	0.002 (2)
C7X	0.013 (2)	0.0213 (18)	0.032 (3)	−0.0016 (14)	0.0023 (18)	0.002 (2)
C8X	0.0144 (16)	0.0268 (17)	0.0247 (19)	−0.0003 (12)	0.0018 (14)	−0.0018 (15)
C9X	0.010 (2)	0.035 (3)	0.023 (3)	0.0040 (18)	0.005 (2)	−0.003 (3)
C10X	0.021 (2)	0.022 (3)	0.051 (4)	0.016 (2)	0.007 (3)	0.001 (3)
C11X	0.0132 (15)	0.052 (2)	0.0276 (19)	0.0019 (14)	−0.0032 (14)	−0.0008 (17)
C12X	0.0193 (15)	0.0391 (19)	0.0294 (18)	−0.0090 (13)	−0.0031 (13)	−0.0029 (14)
C13X	0.0243 (16)	0.0254 (16)	0.0302 (18)	−0.0062 (12)	0.0011 (13)	−0.0025 (12)
N1	0.0155 (6)	0.0161 (6)	0.0264 (6)	0.0011 (4)	0.0012 (5)	0.0000 (4)
N2	0.0220 (7)	0.0179 (6)	0.0526 (9)	0.0009 (5)	−0.0147 (6)	−0.0074 (6)
C1	0.0171 (7)	0.0200 (7)	0.0251 (7)	0.0015 (5)	0.0012 (5)	−0.0020 (5)
C2	0.0165 (7)	0.0285 (8)	0.0253 (7)	0.0031 (6)	0.0008 (5)	−0.0008 (6)
C3	0.0232 (8)	0.0318 (9)	0.0356 (9)	0.0091 (6)	0.0016 (6)	0.0068 (7)
C4	0.0313 (9)	0.0191 (7)	0.0563 (11)	0.0053 (6)	0.0030 (8)	0.0057 (7)
C5	0.0231 (8)	0.0179 (7)	0.0366 (8)	−0.0001 (6)	0.0068 (6)	0.0006 (6)
C6	0.0254 (9)	0.0189 (7)	0.0652 (12)	−0.0044 (6)	0.0036 (8)	−0.0039 (7)

### Geometric parameters (Å, º)

**Table:**

Cl1—C10	1.753 (7)	C11X—C12X	1.385 (5)
O1—C7	1.253 (6)	C11X—H11B	0.9500
O2—C7	1.258 (14)	C12X—C13X	1.383 (4)
C7—C8	1.509 (8)	C12X—H12B	0.9500
C8—C9	1.372 (12)	C13X—H13B	0.9500
C8—C13	1.390 (5)	N1—C1	1.3530 (18)
C9—C10	1.413 (16)	N1—C5	1.3599 (19)
C9—H9A	0.9500	N1—H2N2	0.94 (2)
C10—C11	1.379 (11)	N2—C1	1.335 (2)
C11—C12	1.385 (6)	N2—H1N1	0.88 (2)
C11—H11A	0.9500	N2—H1N2	0.86 (2)
C12—C13	1.390 (4)	C1—C2	1.4067 (19)
C12—H12A	0.9500	C2—C3	1.363 (2)
C13—H13A	0.9500	C2—H2A	0.9500
Cl1X—C10X	1.747 (6)	C3—C4	1.398 (2)
O1X—C7X	1.259 (7)	C3—H3A	0.9500
O2X—C7X	1.253 (14)	C4—C5	1.365 (2)
C7X—C8X	1.519 (8)	C4—H4A	0.9500
C8X—C9X	1.389 (13)	C5—C6	1.503 (2)
C8X—C13X	1.395 (5)	C6—H6A	0.9800
C9X—C10X	1.377 (17)	C6—H6B	0.9800
C9X—H9XA	0.9500	C6—H6C	0.9800
C10X—C11X	1.388 (12)		
			
O1—C7—O2	123.8 (9)	C10X—C11X—H11B	121.4
O1—C7—C8	117.2 (6)	C13X—C12X—C11X	120.3 (3)
O2—C7—C8	118.9 (8)	C13X—C12X—H12B	119.9
C9—C8—C13	118.3 (6)	C11X—C12X—H12B	119.9
C9—C8—C7	121.2 (6)	C12X—C13X—C8X	120.4 (3)
C13—C8—C7	120.5 (4)	C12X—C13X—H13B	119.8
C8—C9—C10	122.8 (10)	C8X—C13X—H13B	119.8
C8—C9—H9A	118.6	C1—N1—C5	122.95 (13)
C10—C9—H9A	118.6	C1—N1—H2N2	120.4 (14)
C11—C10—C9	117.1 (7)	C5—N1—H2N2	116.7 (14)
C11—C10—Cl1	124.2 (7)	C1—N2—H1N1	117.5 (14)
C9—C10—Cl1	118.7 (8)	C1—N2—H1N2	121.7 (14)
C10—C11—C12	121.2 (4)	H1N1—N2—H1N2	119 (2)
C10—C11—H11A	119.4	N2—C1—N1	117.63 (13)
C12—C11—H11A	119.4	N2—C1—C2	123.89 (14)
C11—C12—C13	120.1 (3)	N1—C1—C2	118.48 (13)
C11—C12—H12A	119.9	C3—C2—C1	119.04 (14)
C13—C12—H12A	119.9	C3—C2—H2A	120.5
C8—C13—C12	120.3 (3)	C1—C2—H2A	120.5
C8—C13—H13A	119.8	C2—C3—C4	120.93 (14)
C12—C13—H13A	119.8	C2—C3—H3A	119.5
O2X—C7X—O1X	127.3 (10)	C4—C3—H3A	119.5
O2X—C7X—C8X	117.5 (8)	C5—C4—C3	119.24 (15)
O1X—C7X—C8X	115.2 (7)	C5—C4—H4A	120.4
C9X—C8X—C13X	120.8 (6)	C3—C4—H4A	120.4
C9X—C8X—C7X	117.9 (6)	N1—C5—C4	119.32 (15)
C13X—C8X—C7X	121.3 (4)	N1—C5—C6	115.76 (13)
C10X—C9X—C8X	116.4 (9)	C4—C5—C6	124.91 (15)
C10X—C9X—H9XA	121.8	C5—C6—H6A	109.5
C8X—C9X—H9XA	121.8	C5—C6—H6B	109.5
C9X—C10X—C11X	124.5 (7)	H6A—C6—H6B	109.5
C9X—C10X—Cl1X	118.1 (8)	C5—C6—H6C	109.5
C11X—C10X—Cl1X	117.3 (7)	H6A—C6—H6C	109.5
C12X—C11X—C10X	117.3 (4)	H6B—C6—H6C	109.5
C12X—C11X—H11B	121.4		
			
O1—C7—C8—C9	169.3 (5)	C7X—C8X—C9X—C10X	172.7 (6)
O2—C7—C8—C9	−9.3 (8)	C8X—C9X—C10X—C11X	6.5 (12)
O1—C7—C8—C13	−11.7 (7)	C8X—C9X—C10X—Cl1X	−175.4 (5)
O2—C7—C8—C13	169.7 (6)	C9X—C10X—C11X—C12X	−4.3 (11)
C13—C8—C9—C10	4.3 (9)	Cl1X—C10X—C11X—C12X	177.7 (4)
C7—C8—C9—C10	−176.7 (6)	C10X—C11X—C12X—C13X	1.0 (7)
C8—C9—C10—C11	−5.7 (11)	C11X—C12X—C13X—C8X	−0.4 (6)
C8—C9—C10—Cl1	177.0 (5)	C9X—C8X—C13X—C12X	2.8 (7)
C9—C10—C11—C12	3.9 (10)	C7X—C8X—C13X—C12X	−175.5 (3)
Cl1—C10—C11—C12	−178.8 (5)	C5—N1—C1—N2	−177.15 (15)
C10—C11—C12—C13	−1.1 (7)	C5—N1—C1—C2	1.9 (2)
C9—C8—C13—C12	−1.2 (6)	N2—C1—C2—C3	176.80 (16)
C7—C8—C13—C12	179.9 (4)	N1—C1—C2—C3	−2.2 (2)
C11—C12—C13—C8	−0.4 (6)	C1—C2—C3—C4	0.7 (3)
O2X—C7X—C8X—C9X	2.7 (9)	C2—C3—C4—C5	1.2 (3)
O1X—C7X—C8X—C9X	−176.6 (5)	C1—N1—C5—C4	0.0 (2)
O2X—C7X—C8X—C13X	−178.9 (6)	C1—N1—C5—C6	−179.36 (14)
O1X—C7X—C8X—C13X	1.8 (6)	C3—C4—C5—N1	−1.5 (3)
C13X—C8X—C9X—C10X	−5.7 (9)	C3—C4—C5—C6	177.75 (17)

### Hydrogen-bond geometry (Å, º)

**Table:**

*D*—H···*A*	*D*—H	H···*A*	*D*···*A*	*D*—H···*A*
N1—H2*N*2···O1	0.94 (2)	1.69 (2)	2.614 (7)	168 (2)
N2—H1*N*2···O2	0.86 (2)	1.98 (3)	2.832 (14)	170 (2)
N2—H1*N*1···O2^i^	0.88 (2)	2.06 (2)	2.853 (11)	150 (2)

Symmetry code: (i) −*x*+1/2, −*y*+1/2, −*z*.
